# Seasonal Transition in the Dominance of Photoautotrophic and Heterotrophic Protists in the Photic Layer of a Subtropical Marine Ecosystem

**DOI:** 10.1111/1758-2229.70126

**Published:** 2025-07-14

**Authors:** Yoav Avrahami, Raffaele Siano, Maxim Rubin‐Blum, Gil Koplovitz, Nicolas Henry, Colomban de Vargas, Miguel J. Frada

**Affiliations:** ^1^ Department of Ecology, Evolution and Behavior Silberman Institute of Life Sciences, The Hebrew University of Jerusalem Jerusalem Israel; ^2^ The Interuniversity Institute for Marine Sciences in Eilat Eilat Israel; ^3^ IFREMER DYNECO Plouzané France; ^4^ Charney School of Marine Sciences University of Haifa Haifa Israel; ^5^ Israel Oceanographic and Limnological Research, National Institute of Oceanography Haifa Israel; ^6^ CNRS, Sorbonne Université, FR2424, ABiMS, Station Biologique de Roscoff Roscoff France; ^7^ Research Federation for the Study of Global Ocean Systems Ecology and Evolution, FR2022/Tara GOSEE Paris France

**Keywords:** diversity, ecology, functional traits, photic layer, protists, Red Sea, subtropical

## Abstract

Protists are major functional players in the oceans. Time‐resolved protist diversity and succession patterns remain poorly described in subtropical ecosystems, limiting current understanding of food web dynamics and responses to environmental changes in these major world‐ocean regions. We used amplicon sequencing data and trait‐based annotation to examine the seasonality of planktonic protists in the subtropical Gulf of Aqaba (Red Sea). Temperature and nutrients were the major drivers of succession. We detected marked seasonal shifts in protists. Heterotrophs, including diverse parasitic functional groups, dominated the warm, stratified oligotrophic period spanning spring and summer. By contrast, nutrient influx during deep convective mixing in winter triggered a shift to photoautotrophic communities dominated by a few genera of chlorophytes. Deeper winter mixing resulted in larger blooms at the onset of stratification dominated by diatoms, relative to chlorophytes that prevailed during shallower blooms. This result illustrates the impact of mixing depth on bloom formation and composition. Comparisons with oceanwide rDNA datasets indicate that the oligotrophic protist assemblages from the Gulf resemble those from warm, open oceans. This work provides a detailed assessment of the seasonal switch in dominant trophic functions in protists in phase with nutrient levels in a subtropical planktonic ecosystem.

## Graphical abstract

Amplicon sequencing showed a distinct seasonality in protist communities and functional strategies in a subtropical ecosystem in the Gulf of Aqaba. Temperature and nutrients were major drivers of succession. Comparisons with oceanwide Tara‐Ocean datasets indicated that the summer‐oligotrophic protist assemblages from the Gulf of Aqaba resemble those from warm, open oceans.

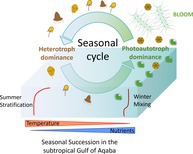

## 1 Introduction

Planktonic protists represent the majority of eukaryotic diversity in the sunlit layer of the oceans, comprising a vast and still largely unknown variety of species and functional roles that structure the marine food web and drive global biogeochemical cycles (de Vargas et al. 2015; Falkowski et al. 2004; Guidi et al. 2016; Lima‐Mendez et al. 2015; Worden et al. 2015). Photoautotrophic protists (eukaryotic phytoplankton) act as the main primary producers forming the base of the marine food web (Behrenfeld et al. 2001), while heterotrophic protists act as links to higher trophic levels by consuming bacteria and phytoplankton while serving as food to larger zooplankton (Sherr and Sherr 2002). However, the dichotomic differentiation between primary producers and microbial consumers has been blurred over the last decades, given that most phytoplankton have been recognised as mixotrophic, being capable of engaging both photoautotrophy and heterotrophy (Mitra et al. 2023). The heterotrophic component occurs via direct absorption of dissolved organics (osmotrophy) or by engulfing particulate organic matter, including other living cells (phagotrophy). Osmotrophy is likely ubiquitous in phytoplankton, while phago‐mixotrophic appears to be common in most phytoplankton groups, with few exceptions such as in diatoms and small chlorophytes (e.g., *Ostreococcus*) (Mitra et al. 2023; Stoecker et al. 2017). Many phytoplankton and other marine protists can establish mutualistic or parasitic associations and act as major regulators of plankton successions (Anderson and Harvey 2020; Bass et al. 2021; Blindheim et al. 2023; Coats 1999). Much protistan diversity has not yet been characterised or cultured, and field studies of protist assemblages complemented by physiological lab experiments are rare (Caron et al. 2009; Keeling 2013; Keeling and del Campo 2017). However, this is critical for a better understanding of the functional basis of marine ecosystems. Over the last decades, high‐throughput amplicon sequencing of biodiversity marker genes (barcodes) has provided accounts of the taxonomic diversity in oceanic samples (de Vargas et al. 2015; Lima‐Mendez et al. 2015; Massana et al. 2015; Meyneng et al. 2024; Pernice et al. 2016). Combined with more recent functional annotation of microbial sequences, it has enabled the advancement of the current understanding of the complexity of food web structure and the role of microorganisms in marine ecosystems (Ramond et al. 2019; Ramond et al. 2024).

Global coverage of protists diversity has vastly expanded following the circum‐global transects of *Tara* Oceans and Malaspina expeditions (de Vargas et al. 2015; Lima‐Mendez et al. 2015; Malviya et al. 2016). However, time‐resolved coverage of subtropical, oligotrophic marine regions that occupy ~40% of the surface of the earth and account for ~25% of the marine primary production (Duarte et al. 2013; Longhurst 1995; Polovina et al. 2008) is still limited (Blanco‐Bercial et al. 2022; Pasulka et al. 2013). Temporal resolution is, however, critical to identifying the patterns of microbial community succession and turnover, as well as identifying core microbiota from these regions and their relationship to other ecosystems. Moreover, global temperature trends indicate that subtropical oceans will expand pole‐wards, with subtropical protists replacing higher‐latitude communities (Cheng et al. 2022; Chust et al. 2014). Therefore, better descriptions of standing‐stock diversity and temporal dynamics in oligotrophic ecosystems are fundamental to model community responses to climate change and generate baseline information to evaluate future shifts in microbial communities.

**FIGURE 1:**
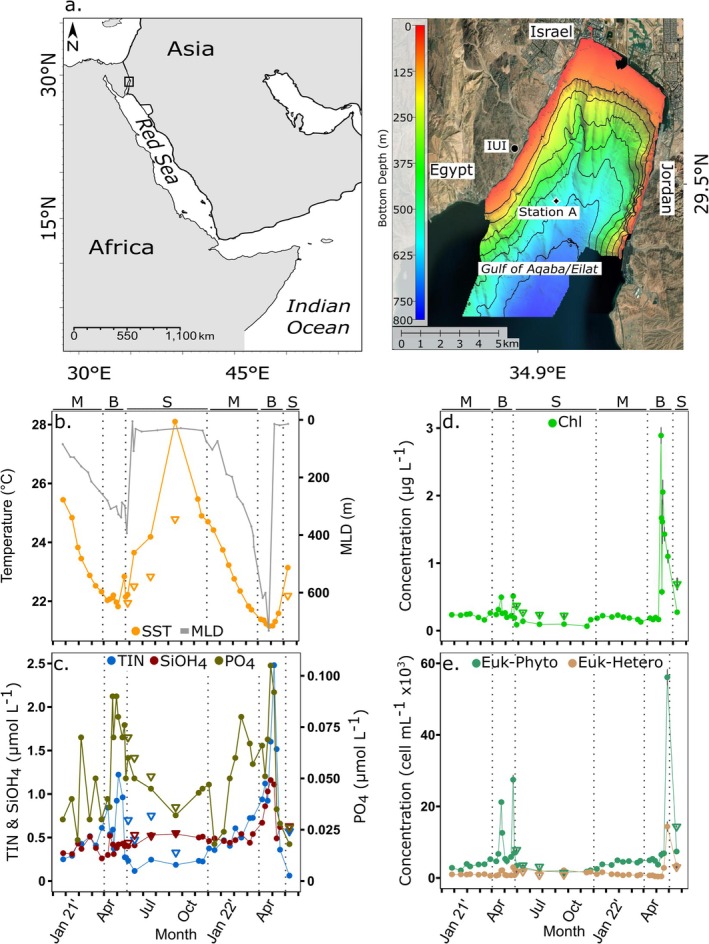
Location and hydrographic variables in the Gulf of Aqaba. (a) Map of the Red Sea and Gulf of Aqaba (GoA). The GoA is located in the northern‐Eastern tip of the Red Sea (indicated in square in the left panel). Sampling in this study was collected at the ‘Station A’, in the northern GoA as depicted in the right‐side panel. The map of the GoA was modified from Israel Oceanographic and Limnological Research and Geological Survey Israel. (b) Temperature at the Sea surface (SST) and DCM, and mixed layer depth (MLD). (c) Macronutrients: Total Inorganic Nitrogen (TIN), Silica and Phosphate. (d) Chlorophyll *a*. (e) Total eukaryotic phytoplankton (Euk‐Phyto) and eukaryotic heterotrophs (Euk‐Hetero) as determined by flow cytometry. Grey bars in C and D represent the standard deviation (*n* = 3, *n* = 2, respectively). Measurements at the Deep chlorophyll maximum (DCM) during the stratified season are represented as triangles. The seasons are separated by dashed lines and noted by letters in the top of each panel: M = mixing, B = bloom, S = summer stratification.

To address this knowledge gap, we examined the diversity and community succession of protists in the photic layer inhabiting the open sea in the subtropical Gulf of Aqaba/Eilat (GoA) in the northern section Red Sea (Figure 1). Although the GoA is a marginal, semi‐enclosed marine basin, the open‐sea areas display seasonally physical and chemical characteristics that can be related and thus provide insights on global dynamics in oligotrophic regions of the oceans. Namely, from about April to November, the upper water column is warm (up to average 28°C) and stratified (with a shallow, typically < 30 m wind‐driven mixed layer). Surface chlorophyll *a* (Chl*a*) concentration is low (< 0.5 μg Chl*a* L^−1^), cyanobacteria numerically dominate the phytoplankton communities, and a deep‐chlorophyll maximum forms at about ~80–100 m deep at the verge of the nutricline (Landou et al. 2023; Lindell and Post 1995; Zarubin et al. 2017). The dominance of cyanobacteria and the recent examination of coccolithophore communities support the resemblance of the stratified season in the GoA to other oligotrophic regions in the ocean (Frada et al. 2022; Keuter et al. 2023). However, conditions change radically during winter to mesotrophic conditions, possibly resembling more coastal settings. This shift in conditions occurs following progressive surface cooling from November to a minimum of about 21°C–22°C around March that drives prevalent deep convective mixing to hundreds of meters and the transport of nitrate and phosphate to the surface layer (Landou et al. 2023; Lindell and Post 1995; Zarubin et al. 2017). Cyanobacteria decline (Lindell and Post 1995), while coccolithophore species (such as *Gephyrocapsa huxleyi*) and diatoms that inhabit nutrient‐richer settings accumulate during winter (Al‐Najjar et al. 2007; Avrahami et al. 2025; Frada et al. 2022; Keuter et al. 2023). Such deep mixing enabled by the characteristic warm intermediate and bottom water layers in the Gulf, can reach between 300 and > 700 m during cold winter (Genin et al. 1995). Deep mixing is halted at the onset of stratification, setting the stage for the formation of phytoplankton blooms of magnitudes (from 1 to > 2 μg Chl*a* L^−1^) that are unusual in oligotrophic regions (Genin et al. 1995; Lindell and Post 1995; Zarubin et al. 2017). Within about 1 month, the phytoplankton blooms utilise most nutrients and decay as the stratified, oligotrophic conditions are reset (Shaked and Fine 2023; Zarubin et al. 2017). In addition to the assessment of oligotrophic communities, the recurrent seasonal cycle in the GoA provides a good opportunity to study protist diversity succession and community responses to environmental gradients and responses to interannual variations in a subtropical marine ecosystem.

Here, we investigate the sucession of protist assemblages from the GoA by rDNA amplicon sequencing of the V4 region of the 18S rRNA gene annotated both taxonomically using curated datasets (Guillou et al. 2013) and with a trait approach to assess the functional diversity of morphological and/or trophic characteristics of organisms (Ramond et al. 2019). We then compared our amplicon dataset to a comparable global marine collected from the *Tara* Ocean expedition (Pesant et al. 2015). This analysis served to test the resemblance of protistan communities from the GoA to other ocean locations and to determine if the photic zone of the GoA can serve as a research model for open‐ocean subtropical ecosystems. Our study provides a detailed synthesis of the seasonal succession patterns of planktonic protists in subtropical marine ecosystems.

## 2 Materials and Methods

### 2.1 Study Site and Sampling

Between November 2020 and May 2022, near‐surface water samples were collected from a depth of ~2 m at the open ocean site ‘station A’ (29°28′ N, 34°55′ E) in the northern GoA (bottom depth ~ 700 m) (Figure 1a). During mixing (November–February), samples were collected twice a month. At the peak of mixing and during the spring bloom (March–April), samples were collected weekly or twice per week to capture the rapid phytoplankton bloom dynamics. At each time point, seawater was transferred into acid‐washed carboys using a manual pump installed in a boat and pre‐filtered by 500 μm mesh to avoid large zooplankton. In addition, a Sea‐Bird SBE 19 CTD (conductivity, temperature, depth and Sea‐Bird Scientific) was deployed to obtain the physical parameters of the water column. In the stratified summer (late April–August) samples were collected both from the surface and the deep chlorophyll maximum (DCM) using 12 L Niskin bottles (General Oceanics) installed on the Sea‐Bird carousel and coupled with Sea‐Bird SBE 19+ CTD (Sea‐Bird Scientific). Mixed layer depth (MLD) was calculated according to Zarubin et al. (2017), as the depth in which there is a difference of 0.2°C of potential temperature compared to 3 m depth. Seawater samples were further processed within 30–60 min of collection.

### 2.2 DNA Extraction and Amplicon Sequencing

Duplicates of ~4.4 L seawater were filtered onto 0.2 μm Sterivex filters (Merck Millipore), flash‐frozen in liquid N_2_ and stored at −80°C until DNA extraction. DNA was extracted using the NucleoSpin Plant II kit (Macherey‐Nagel), following the protocol used by the *Tara* Oceans project (Alberti et al. 2017). The extraction began with incubation of the filter with proteinase K and lysozyme (120 min at 56°C). Lysate was transferred to a clean tube and was treated according to the kit's guidelines. Blank samples of clean filters were used as negative controls. Following the extraction, DNA quality and concentrations were measured with the QFX fluorometer (Denovix), using the dsDNA quantification assay (high sensitivity). After DNA extraction and quality test, samples were shipped to the Genomics and Microbiome Core facility at Rush University for PCR and sequencing by MiSeq v2 chemistry (Illumina). Primers targeted the V4 domain of 18S rDNA (~380 bp), as used by the *Tara* Ocean project (TAReuk. Forward: 5′‐CCAGCASCYGCGGTAATTCC‐3′, Reverse: 5′‐ACTTTCGTTCTTGATYRA‐3′) (Stoeck et al. 2010). Sequence data are available at GenBank (accession PRJNA1169359).

### 2.3 Sequence Data Processing and Analyses

Amplicon sequences were processed with Qiime 2 (Bolyen et al. 2019). Demultiplexed paired‐end reads were trimmed with Cutadapt (Martin 2011) without indels, to remove primer sequences, followed by quality control and denoising by DADA2 (Callahan et al. 2016). Sequences were assigned to ASVs and annotated by the protist reference database PR^2^ (version 4.14) (Guillou et al. 2013) using BLAST with a cutoff of 90% (Camacho et al. 2009). 7370 unique ASVs were identified after the removal of unassigned reads and annotations of the phylum Metazoa. Duplicates per each sampling point were averaged and yielded 43 sampling points, which included 1,180,541 reads in total. Saturation of taxa diversity of over 99.9% in all sampling points was verified by rarefaction curves with the R package vegan (Oksanen et al. 2022; R Core Team 2021) (Figure S1a).

Richness was calculated based on the ACE index, which distinguishes rare and abundant species in its formulation with the R package phyloseq (McMurdie and Holmes 2013). Turnover (temporal beta diversity index) was calculated with the R package codyn (Hallett et al. 2016) as species appearance and disappearance divided by total species between two time points. This analysis was performed on one time point only for each month to avoid bias of different time intervals between sampling points.

To compare the GoA and *Tara* Oceans datasets, we used the ‘Tara Oceans (2009‐2013) rDNA 18S V4 ASV table’ (Delage et al. 2023), extracting only samples comprising full size‐fraction (> 0.8 μm). This resulted in *Tara* samples from the North and South Atlantic and Pacific and one sample from the mid‐Red Sea collected during winter. Community composition explained by environmental drivers was based on relative abundance values and carried by Redundancy Analysis (RDA) with the vegan package in R (Oksanen et al. 2022). RDA analysis of the merged datasets was performed at the taxon level (based on PR^2^ version 5), meaning that ASVs with similar taxonomic assignments were grouped together and considered as one taxon.

Trait data curated by (Ramond et al. 2019) encompassed morphology, physiology, life‐cycle and trophic mode of protists and covered 2006 taxa with 32 traits. We selected the 18 most informative traits and manually assigned taxa in our study on the genus level, as protistan functional traits are overall conserved within genera (Finlay 2004; Litchman et al. 2007; Violle et al. 2011). When species of the same genus showed different values of a trait (e.g., had different symmetry), the trait was assigned as NA. Functional assignment in the GoA applied for 243 genera (average 80% of the total genera in the GoA). Then, based on Gower distance method (Gower 1971), we clustered genera with similar trait composition into functional groups. The number of groups was determined based on K‐means clustering and Principal Coordinate Analysis (PCoA) which placed the selected genera based on their trait similarity on an ordination space and highlighted 8 distinct groups. Next, we characterised each group based on the main shared traits and defined the groups according to size and trophic mode, and to a lesser extent characteristics of cell cover. The obtained groups were: pico/nano‐autotrophs, silicified‐autotrophs, micro‐autotrophs, pico‐parasite, pico‐heterotrophs (including different heterotrophs as phagotrophs, parasites, saprotrophs and osmotrophs), pico‐phagotrophs, micro‐phagotrophs and armoured‐micro‐phagotrophs. We note that phago‐mixotrophy was not included as a single trophic mode in the functional‐trait database. However, phago‐mixotrophy likely prevails among many species included in the pico/nano‐autotroph and micro‐autotroph functional groups like for example *Teleaulax* (Cryptophyta) (Yoo et al. 2017), dinoflagellates such as for example *Gonyaulax*, *Torodinium* and *Tripos* (Gómez 2009; Jeong et al. 2005; Lambert et al. 2022; Smalley et al. 2003) and haptophytes (Avrahami and Frada 2020; Unrein et al. 2014). In the discussion we will refer to phago‐mixotrophy because of its likely importance during oligotrophic periods.

Functional richness index (FRic) was used to assess the diversity of traits within a community of organisms. It refers to the range of phenotypic variation of traits that assemblage of species can display. The range of phenotypic variation is measured as the volume of a convex hull formed by the presence of phenotypes in an ordination space. The convex hull is the smallest convex polygon that encloses all the points representing the species in a multidimensional functional space (where each dimension represents a functional trait). A larger volume indicates greater functional richness (Villéger et al. 2008). In this study, it was calculated by using the R package mFD (Magneville et al. 2022). We compared functional richness to genera richness (Figure 5), hence the same data of 243 selected genera was used for this analysis (and not total ASV's as used to measure taxonomic richness).

To compare the functional composition of the GoA with the global *Tara* Ocean data, we assigned additional genera that were highly represented by total reads in *Tara* Ocean stations (and were absent or in low read abundance in the GoA). We obtained a total of 432 genera from both GoA and *Tara* data sets, which represented over 70% of total reads per station. Following the same clustering protocol, 6 groups emerged similar to the GoA‐only functional groups, whereas the smaller number of groups stemmed from the merging of several size classes into groups of a wider size range. The outcome groups were: pico‐nano‐micro‐autotrophs, silicified‐autotrophs, pico‐parasite, pico‐phagotrophs, nano‐micro‐phagotrophs and armoured‐micro‐phagotrophs.

### 2.4 Analyses of Nutrients and Chlorophyll a Concentrations

Total inorganic nitrogen (TIN, NO_2_ + NO_3_ + NH_4_), silicate (SiO_2_) and orthophosphate (PO_4_) were determined. For the analysis of NO_2_, NO_3_, silicate and phosphate, sub‐samples of 10 mL were transferred in triplicates to 15 mL Falcon tubes and analysed with Flow Injection autoanalyser (Quik‐Chem 8500, LACHAT Instruments) (Grasshof et al. 2009; Strickland and Parsons 1972). Every run was calibrated by using commercial standards with at least 5 sub‐standards to cover the whole working range. For ammonium (NH_4_) measurements, quadruplicates of 4 mL were transferred to 15 mL Falcon tubes and analysed fluorometrically (Hoefer).

Chlorophyll a was determined following a standard protocol (Jeffrey and Humphrey 1975). Briefly, triplicates of 300 mL seawater were filtered onto Whatman glass fibre filters (GF/F, 25 mm diameter, 0.7 μm pore size). The filters were incubated in 90% acetone (Carlo Erba Reagents) buffered with saturated MgCO_3_ (Sigma–Aldrich), over‐night (4°C in darkness), and analysed fluorometrically for Chl*a* (Trilogy, Turner Designs).

### 2.5 Cell Counts by Flow Cytometry

Seawater samples (triplicates, 4 mL) for enumeration of phototrophic pico‐ and nano‐eukaryotes and heterotrophic pico and nano eukaryotes were collected in sterile Falcon tubes and preserved with 0.25% glutaraldehyde. Samples were flash‐frozen in liquid nitrogen and stored at −80°C until analysis. Cell counts were undertaken on an Attune NxT flow cytometer (Life Technologies) with a 488 nm laser. Each 4 mL sample was subsampled for three protocols: (1) Phototrophic cells were counted for 3 min at a flow rate of 100 μL min^−1^. Populations were distinguished using forward scatter and Chl*a* fluorescence (694/40 bandpass filter) (Marie et al. 1999). (2) Samples for the enumeration of heterotrophs were stained with SYBR Green I (final concentration 1:10,000) and dark incubated for 15 min before cells were counted for 5 min at a flow rate of 100 μL min^−1^. Cells were distinguished based on Chl*a* fluorescence (694/40 bandpass filter) and green fluorescence (530/30 bandpass filter) as described in (Christaki et al. 2011).

## 3 Results

### 3.1 Physical, Chemical and Biological Variations Across Seasons

The annual dynamics of environmental parameters in the photic layer of the GoA reflected the typical annual mixing‐stratification cycle described in the introduction (Shaked and Fine 2023; Zarubin et al. 2017). Three annual periods were defined (Figure 1b–c): (1) the stratification period from late April to October, (2) the winter‐mixing period from November to February and (3) the bloom period at the onset of stratification during March and April.

The stratified period, was characterised by progressive higher surface temperature and lower concentrations of nitrate (0.19 μmol L^−1^) and Chl*a* (< 0.14 μg L^−1^). A DCM with higher nutrients and Chl*a* concentrations, was detected between 55 and 100 m deep. The winter mixing period, was characterised by a progressive decline of sea‐surface temperature, erosion of the DCM and an increase in nitrate (0.47 μmol L^−1^) and Chl*a* (0.21 μg L^−1^) concentrations. Finally, the bloom period immediately followed the deepest mixing depth and nutrient peaks and encompassed the development and decay of the annual Chl*a* maxima during the onset of stratification (March–April). The mixing depth reached about 300 m deep in 2021 and surpassed 700 m in 2022. As a consequence, the concentrations of nitrate (1.3 μmol L^−1^), silicate (0.85 μmol L^−1^) and Chl*a* (1.1 μg L^−1^) in April 2022 were much higher than in April 2021 (0.67 μmol L^−1^ nitrate, 0.4 μmol L^−1^ silicate and 0.3 μg L^−1^ Chl*a*).

Variations in the concentration of total eukaryotic pico‐ and nano‐phytoplankton (Euk‐Phyto) measured by flow cytometry closely matched the Chl*a* profiles (Figure 1e). At the sea surface during the stratified period, we detected about 3000 cells mL^−1^. The concentrations increased to about 3870 cells mL^−1^ with the onset of winter mixing and peaked during the blooms together with a peak in Chl*a*. Higher cell counts for Euk‐Phyto were detected in the 2022 bloom (> 12,000 cells mL^−1^) relative to 2021 (> 10,000 cells mL^−1^). Total eukaryotic heterotrophs (Euk‐Hetero) concentrations were considerably lower than photoautotrophs at all times (Figure 1e). A decrease of more than half in concentration was detected through the winter mixing compared to the stratified seasons at the surface, with cell concentrations averaging 966 and 2177 cells mL^−1^, respectively. However, the peak in concentration of Euk‐Hetero coincided with the 2022 bloom, with cells reaching 2840 cells mL^−1^, whereas the 2021 bloom contained relatively lower heterotrophs below concentrations of the stratified season (1307 cells mL^−1^).

### 3.2 Protist Alpha Diversity Patterns and Turnover

**FIGURE 2:**
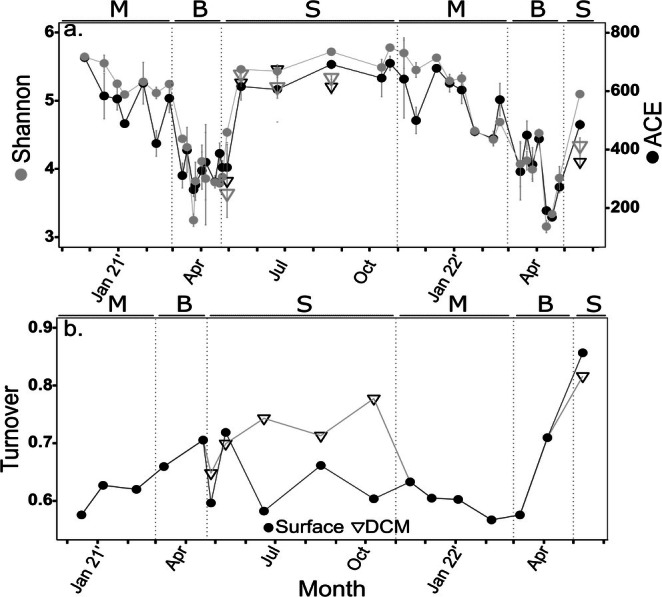
Protist diversity indices. (a) Diversity (Shannon index) and richness (ACE index) of total ASV reads. Grey bars represent standard deviation (*n* = 2). (b) Temporal turnover index. This index detects differences between time points in appearance and disappearance of ASV's. Circles represent samples from sub‐surface waters (2 m depth), while triangles represent samples from the deep chlorophyll maximum (DCM). The seasons are separated by dashed lines and noted by letters in the top of each panel: M = mixing, B = bloom, S = summer stratification.

Total protist richness (ACE Index) and diversity (Shannon Index, H′), based on the ASV reads of the 18S rDNA gene marker, covaried across seasons (Figure 2a). Higher richness and diversity were detected during the stratified season (ACE ~700; H′ > 5) and gradually declined during the mixing period to reach minimal levels during the bloom (ACE < 200; H′ < 4). Rapid recovery in both richness and diversity was detected during the stratified period.

In addition, we determined the turnover index as an estimator of the rate of ASV replacement over time (Figure 2b). Higher turnover rates were detected during the progression of the bloom period, notably in 2022, and particularly at the DCM during the stratified season (0.81 on average). Lower turnover values occurred in surface waters during the stratified season (0.77 on average) and the mixing season (0.72 on average).

### 3.3 Seasonal Variations in Protist Diversity

**FIGURE 3:**
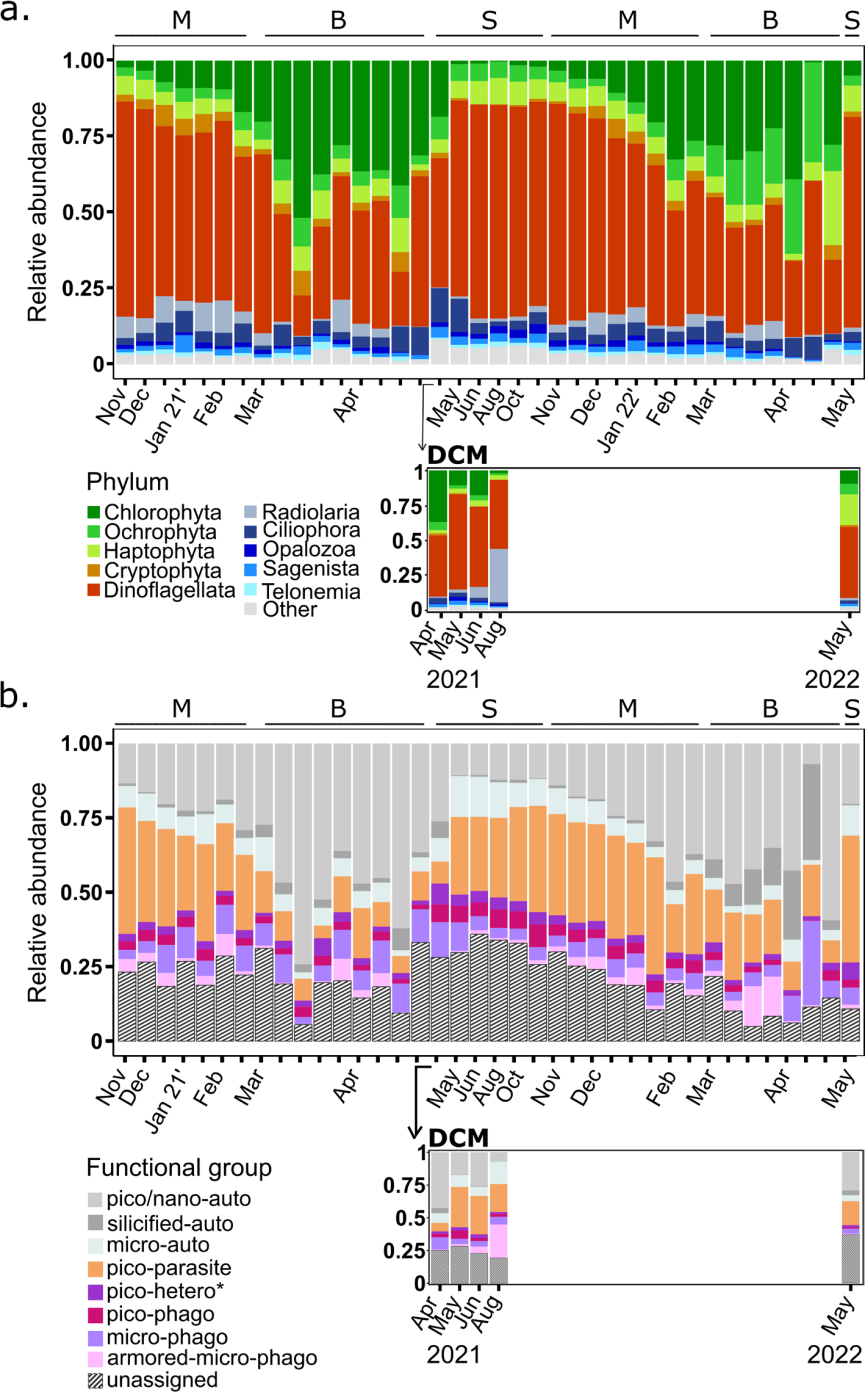
(a) Main Protist phyla in the Gulf of Aqaba. Averaged (duplicates) relative abundance of the top 10 most abundant phyla in surface waters (upper panel) and the corresponding main phyla at the DCM during summer (lower panel), based on ASV's average reads (duplicates). (b) Composition of protistan functional groups over seasonal gradients in the Gulf of Aqaba. Grey‐shaded bars represent autotrophic groups, and coloured bars represent heterotrophic groups. Averaged (duplicates) relative abundance of functional groups at surface waters (upper panel) and the corresponding functional groups at the DCM during summer (lower panel), based on 243 most abundant genera in the GoA. Letters in the top panels represent seasonal periods: M = mixing, B = bloom, S = summer stratification.

**FIGURE 4:**
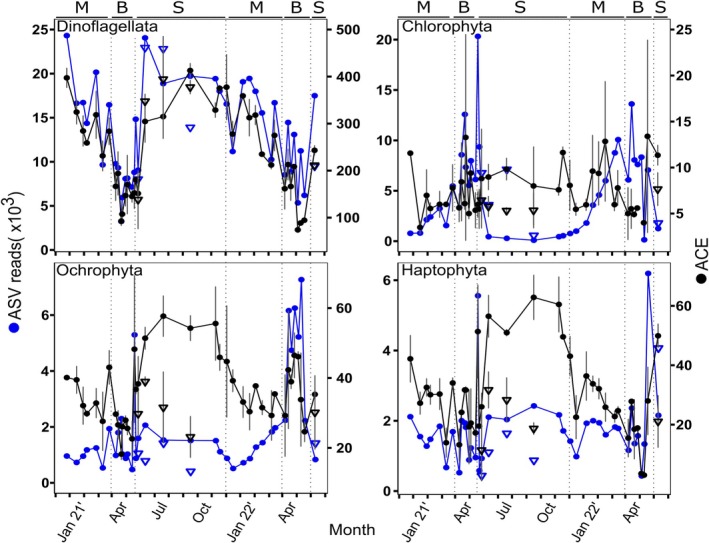
Total ASV reads (blue) and richness (ACE index, black) for main autotrophic phyla: Dinoflagellata, Chlorophyta, Ochrophyta and Haptophyta. Grey bars represent standard deviation (*n* = 2). Circles represent samples from sub‐surface waters (2 m depth), while triangles represent samples from the deep chlorophyll maximum (DCM). The seasons are separated by dashed lines and noted by letters in the top of each panel: M = mixing, B = bloom, S = summer stratification.

Regarding ASV read abundances, Dinoflagellata and Chlorophyta were the most predominant protist phyla, interchanging in relative prevalence between seasons (Figure 3a). Dinoflagellata accounted for up to 65% of the total ASV reads during the stratification period and progressively declined during the mixing and bloom periods to about 30% of total ASV reads. A rapid recovery to high read proportions occurred after the bloom period. Absolute read count and richness for Dinoflagellata were both higher during the stratified period and lower during the bloom (Figure 4). Among Dinoflagellata, ~36% of the ASV reads belonged to undefined Dinophyceae (Dinophyceae_XXX), ~25% to Syndiniales Dino‐groups, and ~10% to a group of dinoflagellate genera composed of *Gyrodinium*, *Prorocentrum* and *Tripos* (Figure S2a). About 24% of Dinoflagellata sequences comprised a variety of taxa annotated at various taxonomic levels (Figure S2a).

By contrast, Chlorophyta accounted for < 5% of the ASV reads at the surface during the stratified season but gradually increased to ~25%–50% through the mixing period peaking during the bloom (Figure 3a). Maxima of Chlorophyta absolute ASV reads and richness were also detected during the bloom (Figure 4). Three single genera prevailed among Chlorophyta: *Ostreococcus, Micromonas* and *Bathycoccus*. *Ostreococcus* represented ~85% of Chlorophyta ASVs during the mixing and bloom periods, while *Micromonas* and *Bathycoccus* represented over 90% of Chlorophyta ASVs at the surface during the stratified season (Figure S2b). Noticeably, the relative contribution in Chlorophyta was lower during the bloom of 2022, coinciding with increased ASV reads from Ochrophyta and Haptophyta. Moreover, at the DCM during the stratified season, the relative contribution of Chlorophyta was higher, and *Ostreococcus* contributed 15%–80% of the Chlorophyta ASV read abundances.

Ochrophyta and Haptophyta represented ~13% of total ASV reads across seasons. A peak of relative abundance was distinct during both annual blooms (Figure 3a). However, in absolute terms, both ASV reads abundance and richness were much higher during the bloom of 2022 (Figure 4). *Aureococcus* and the diatoms *Skeletonema, Thalassiosira* and other species from the Family Mediophyceae were the most common Ochrophyta during the later mixing period and bloom (Figure S2c). *Pelagomonas* prevailed during the early mixing and DCM. Representatives of the Marine Ochrophyta group (MOCH‐2; Massana et al. 2014) and a large fraction of unannotated sequences (> 65%) prevailed in surface waters during the stratified period and the early mixing (Figure S2c). The genera *Chrysochromulina* and the coccolithophore *Gephyrocapsa* were the most common Haptophyta, namely during the 2022 bloom. Cryptophyta represented < 5% of the total ASV reads during the mixing and bloom season and were rarer during the stratified season.

Finally, heterotrophic protists (although various can carry symbiotic partners) represented 10%–25% of the ASV reads across all time points. Among heterotrophs, Radiolaria, Ciliophora and Opalozoa represented the most abundant phyla. Radiolaria were more prevalent during winter (except for a peak in August at the DCM). Ciliophora peaked during the bloom and the early stratified period (Figures 3a and S2d,e).

### 3.4 Seasonal Variation in Functional Groups of Protists

Analyses of the functional traits of most of the protist ASVs with clear taxonomic identification enabled the definition of eight functional clusters (Figures 3b and S3): (1) pico/nano‐autotrophs, composed mainly of small chlorophytes and other small phytoplankton groups; (2) silicified‐autotrophs, composed of diatoms; (3) micro‐autotrophs, composed mainly of dinoflagellates; (4) pico‐parasites, composed of Syndiniales and other Dinophyceae; (5) pico‐heterotrophs as Picozoa and Telonemia corresponding to a variety of functional types including osmotrophic, parasitic and saprotrophic organisms; plus three groups of heterotrophic genera with predatorial lifestyles differentiated based on size; (6) pico‐phagotrophs, composed of heterotrophic Marine Stramenopiles (MAST); (7) micro‐phagotrophs, containing a variety of ciliates and heterotrophic dinoflagellates; and (8) armoured‐micro‐phagotrophs, composed of Radiolaria (Table S1).

Pico/nano‐autotrophs and pico‐parasites were the most prevalent functional groups identified in terms of ASV reads (Figure 3b), broadly matching the contrasting seasonality of Chlorophyta and parasitic dinoflagellates (Syndiniales and other parasitic taxa), respectively (Figure S2). The read abundance of the pico/nano‐autotrophs increased during the mixing and bloom, and the read abundance of pico‐parasites increased in the stratified season. The read abundance of micro‐autotrophs also increased during the stratified period. Silicified autotrophs were more common during the bloom, especially in 2022, when they reached 36% of the total read abundance (compared to 8% during the bloom of 2021). Pico‐heterotrophs, pico‐phagotrophs, micro‐phagotrophs and armoured‐micro‐phagotrophs inter‐varied in density over seasons, but as a unit, represented typically 15%–20% of the functional diversity in the GoA. Noticeably, an important fraction of the diversity remained unassigned to any functional category. These represented 5%–20% of the diversity during the bloom and 20%–30% during the stratified and mixing seasons (Figure 3b).

**FIGURE 5:**
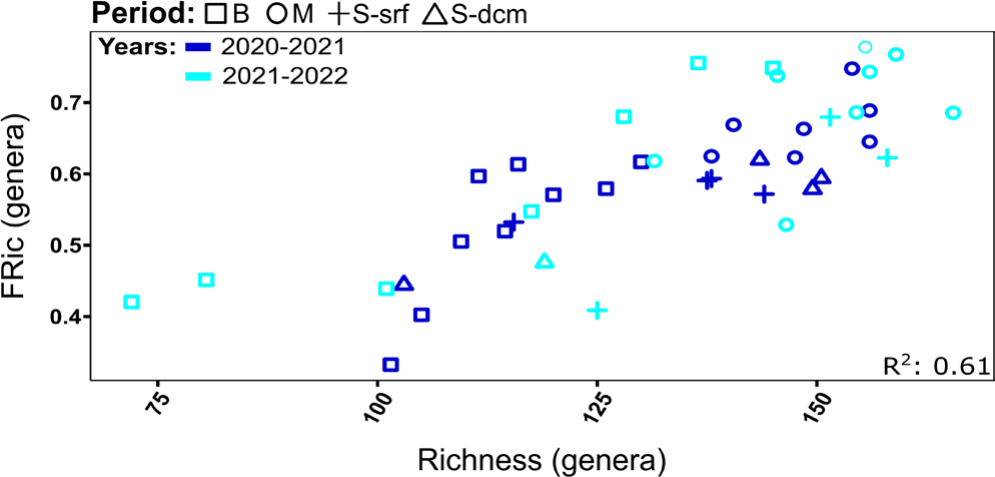
Patterns of taxonomic versus functional richness. Each sampling point was measured for taxonomic richness (*x*‐axis) and functional richness (Fric, *y*‐axis), based on an analysis performed at the genus taxonomic level and including the 243 top protist genera in the GoA. Shapes represent different seasonal periods: Square = bloom, Circle = mixing, Plus = summer‐surface, Triangle = summer‐DCM. Colours represent sampling years: Blue = 2020–2021, cyan = 2021–2022. *R* ^2^ was calculated by a linear model of the 2 variables.

We used the FRic index to assess the extent of functional richness at each sampling point and determine the relation between taxonomic and functional diversity (Villéger et al. 2008). A positive correlation between both the functional and taxonomic richness was detected (*R* ^2^ = 0.61, Figure 5). Time points from the mixing and stratified seasons displayed the highest genetic and functional diversity, while by contrast, bloom time points had overall lower values, although a wider richness range.

### 3.5 Relation Between the GoA and Other Oceanic Regions

**FIGURE 6:**
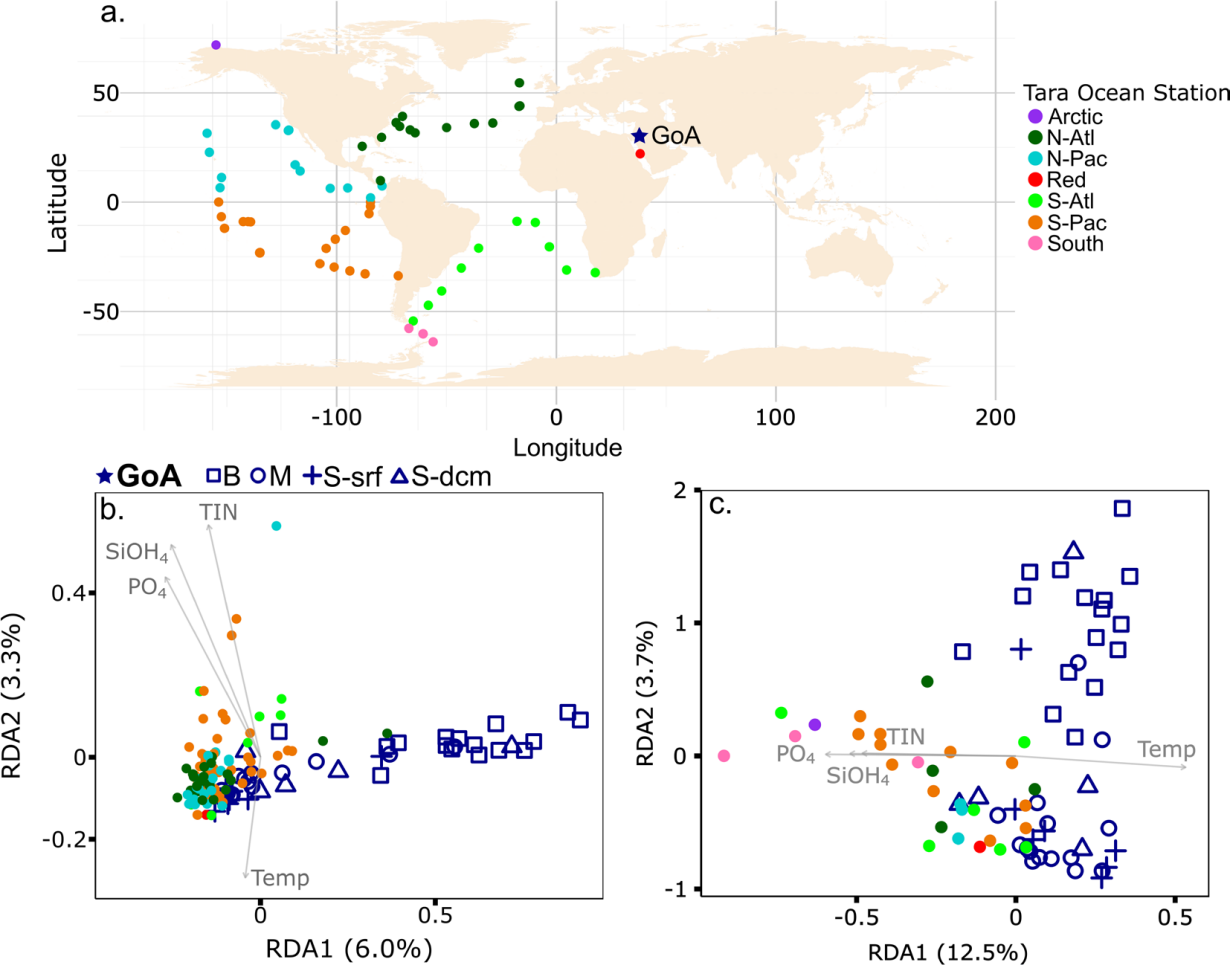
Global‐scale community composition of the GoA and *Tara* Oceans samples. (a) Geographical location of the GoA and *Tara* stations that were examined. RDA analysis was based on the average (duplicates) relative abundance of (b) genera (or lower taxonomic resolution when genus was not assigned) and (c) functional groups at each sampling point. Shapes correspond to the GoA's seasonal period: B = bloom, M = mixing, S‐srf = summer‐surface, S‐dcm = summer‐DCM. Filled circles represent different oceanic basins and seas covered by the *Tara* Oceans cruise: Arctic = Arctic Ocean, N‐Atl = North Atlantic, N‐Pac = North Pacific, Red = Red Sea, S‐Atl = South Atlantic, S‐Pac = South Pacific, South = Southern Ocean. Grey arrows show influential environmental variables (*p* value < 0.05): TIN = total inorganic nitrogen, SiOH_4_ = silica, PO_4_ = phosphate, Temp = temperature. Percentage in parenthesis refers to the explained variability of environmental parameters by each axis.

We integrated our data from the GoA into the amplicon dataset (V4 region of the 18S rDNA) from the *Tara* Oceans expedition (Pesant et al. 2015) to determine how our seasonal periods in the GoA related to open‐ocean across major oceanic basins in terms of protist assemblages (Figure 6a). Sites from the Indian Ocean are missing because those were fractioned by size classes during sampling during the *Tara* expedition and could not be easily compared to our non‐fractionated data set.

We observed a higher dispersion in our seasonal samples from the GoA, which spread along the RDA1 axis (this axis explained 6% of the variability, whereas RDA2 explained 3.3% (Figure 6b). Among the GoA variability, samples collected in surface waters during the stratified season, and some from the DCM, as well as early mixing periods, were associated with samples collected globally in warm, oligotrophic subsurface and DCM waters from the North Atlantic and North and South Pacific oceans. By contrast, most samples from the GoA collected during the late mixing and bloom periods did not cluster with any of the *Tara* Oceans samples from the world's open ocean (Figure 6b). These samples contained a large prevalence of haptophytes from the genus *Chrysochromulina* in the GoA (Figure S4).

To test the relationship in functional diversity of protists in the GoA relative to other oceanic settings, we selected a variety of open‐ocean and oligotrophic regions that showed high taxonomic similarity to the GoA from the *Tara* Oceans dataset and also included additional representative *Tara* Oceans nutrient‐rich stations from the Arctic and Southern Oceans (Figure 6c). Additionally, we annotated the main genera of these stations based on the trait table as described above, and then created a new set of six functional groups that encompassed both *Tara* Oceans and the GoA datasets (Table S2). RDA analysis of the functional group composition revealed that environmental drivers (water temperature and macronutrients) accounted for about 16% of the distance between assemblages (Figure 6c). Again, samples from the GoA mixing and stratified surface water periods were similar in their functional assemblage to open‐ocean stations associated with high water temperatures and low nutrients. Bloom samples were dissimilar to other ocean regions in functional structure and were not strongly associated with water temperature or macronutrients.

## 4 Discussion

We detected diverse planktonic protist communities and marked seasonal differences in protist functional groups between oligotrophic and mesotrophic seasons in the photic layer of the GoA. Diversity differences were also detected between annual phytoplankton bloom, which related with the mixing depth and nutrient availability in the photic layer. Below, we discuss the structure of contrasting protist assemblages between seasons and provide insights into the ecological implications of our findings.

### 4.1 Seasonal Heterotrophic and Autotrophic Functions

Warmer, nutrient‐limited conditions during the stratified season in the GoA largely favoured heterotrophic protist taxa. The establishment of communities dominated by heterotrophic functional types occurred rapidly following the establishment of stratification and nutrient depletion after the phytoplankton bloom declined and persisted through summer. Among heterotrophic groups, a vast diversity of pico‐parasites affiliated to the Syndiniales from the phylum Dinoflagellata was detected, contributing up to 43% of total protist ASV reads during this seasonal period. The Syndiniales is a hyper‐diverse order of endoparasitoids comprising five taxonomic clades (Clade I to V; Guillou et al. 2008; Rizos et al. 2023) that typically infect host cells as minute motile spores (dinospores), multiply intracellularly and finally kill the host before release (Chambouvet et al. 2008; Coats and Park 2002; Decelle et al. 2022). We detected all five clades of Syndiniales in the GoA, but clade I and II were the most prevalent, as in several other marine ecosystems (Anderson et al. 2024; Nagarkar and Palenik 2023; Siano et al. 2011). Both clades can infect various microbial and metazoan hosts (Anderson et al. 2024; Zamora‐Terol et al. 2020). Syndiniales infections can propagate during phytoplankton blooms (Chambouvet et al. 2008). However, in the GoA, Syndiniales diversity and abundance peaked during the stratified season when the density of other microbial cells, including phytoplankton, was the lowest, which plausibly resulted from high host‐specificity that appears to be common in Syndiniales (Chambouvet et al. 2008). Known Syndiniales hosts included other dinoflagellates such as *Gymnodinium*, *Prorocentrum* and *Tripos* (Anderson et al. 2024; Christaki et al. 2023; Nagarkar and Palenik 2023; Siano et al. 2011) and possibly taxa from the very common Dinophyceae_XXX (Nagarkar and Palenik 2023), all of which occurred during the stratified season in the GoA. We note that although important, the contribution of Dinoflagellata (including Syndiniales) during the stratified season is likely inflated due to the usual high number of rDNA copies in their genomes (Anderson et al. 2024; Santoferrara 2019). Still, variations in ASV densities of Syndiniales and high diversity over the annual cycle reflect a real seasonal oscillation in parasitic density with higher frequency during the oligotrophic period. Similar results have been reported in other open‐ocean locations, emphasising the wide prevalence and importance of these parasites in marine ecosystems (Anderson et al. 2024; de Vargas et al. 2015; James et al. 2022; Nagarkar and Palenik 2023; Ollison et al. 2021; Rizos et al. 2023).

In addition, during the stratified season, we detected an important contingent of phagotrophic functional groups. Phagotrophs included namely Ciliophora, MAST and dinoflagellates from the genus *Gyrodinium*, but also many pico/nano autotrophs and micro‐autotrophs with phago‐mixotrophic capabilities such as particularly haptophytes and dinoflagellates from the genera *Gonyaulax*, *Torodinium* and *Tripos* (Gómez 2009; Jeong et al. 2005; Lambert et al. 2022; Smalley et al. 2003; Unrein et al. 2014). All likely benefited from high abundances of heterotrophic bacteria and cyanobacteria during the same period.

By contrast, the onset of the mixing season and increased nutrient supply during the fall triggered the gradual transition to photoautotrophic‐dominated communities. Over this period, the relative prevalence of parasites and phago‐mixotrophs declined, likely reflecting specific host decline (although diverse assortments of Syndiniales remained, suggesting continued parasitic propagation) and plausible out‐competition by photoautotrophic taxa capable of rapid nutrient uptake and growth. Among the latter, small‐sized pico and nano‐autotrophs increased in density, particularly the chlorophyte *Ostreococcus*, such that during the bloom, they accounted for over 50% of the protist ASVs. The overall increment in chlorophytes over the winter validates previous pigment ana HPLC analyses in the GoA (Al‐Najjar et al. 2007), while the dominance of *Ostreococcus* is consistent with an overall preference of this taxa for nutrient‐richer conditions and ecological association to high phytoplankton productivity (Choi et al. 2020; Eckmann et al. 2024; Treusch et al. 2012). Other genera from the order Mamiellales (*Micromonas* and *Bathycoccus*) were relatively more important during the stratified season, with *Ostreococcus* still dominating in deeper and nutrient‐rich DCM layers. This result highlights a consistent differentiation between the main Mamiellales genera and the potential role of phytoplankton standing stocks inhabiting DCM as a reservoir for winter assemblages (Eckmann et al. 2024).

*Ostreococcus* remained the most common ASV during the 2021 bloom. However, during the 2022 bloom, which developed following a deeper mixing and higher nutrient influx, Ochrophyta was much more important. Dominant Ochrophyta included centric diatoms from the genus *Skeletonema* and other polar‐centric‐Mediophyceae taxa. Given the large size of diatom cells relative to *Ostreococcus*, diatom biomass likely over‐dominated primary productivity during this bloom, which is consistent with earlier pigment‐based estimates indicating that diatoms can represent about 40% of the chlorophyll biomass during the bloom in the GoA (Al‐Najjar et al. 2007). However, we note a contrast between the sequence‐based detection of diatoms and earlier microscopy‐based analyses over the same time points (Avrahami et al. 2025). Microscopy‐based analyses indicated that Thalassiosiraceae and large chain‐forming *Chaetoceros* were the most common during blooms. *Skeletonema* was rare by microscopy. The contrast between microscopy and sequencing datasets possibly highlights difficulties in morphologically identifying some taxa or poor annotation of diatom ASVs, which should be improved in future work.

Following the decline of diatoms, we detected a rapid peak in Haptophyta was detected. This haptophyte peak comprised mostly of the coccolithophore *Gephyrocapsa huxleyi*. The sequential rise of diatoms and coccolithophores in response to high nutrient availability is consistent with the *r*‐selected nature of these taxa capable of thriving in high nutrient conditions. Moreover, it resembles a short‐lived version of the classical phytoplankton community succession patterns observed in the North Atlantic, where diatom blooms during early winter deplete silica, which precipitates their decline, after which prymnesiophytes bloom (Barlow et al. 1993). As such, it is plausible that the rapid demise of the diatom bloom in the GoA reflects silica depletion, enabling the growth of succeeding phytoplankton communities. Moreover, the emergence of diatoms and coccolithophores correlated in the two studied years in the GoA with the protist diversity‐minima (Figure 2a). This diversity minimum suggests strong competitive exclusion mechanisms influencing the composition of protist communities as described during phytoplankton dynamics in nutrient‐rich, high latitude regions (Cascella et al. 2020; Choi et al. 2020; Tréguer et al. 2018). In conjunction, blooms were accompanied by peak densities in heterotroph cells, suggesting additional top‐down control of bloom decline. The concerted importance of abiotic and biotic controls, which may also include viruses that are important agents of diatom‐bloom mortality (Kranzler et al. 2019), remains to be studied in the GoA.

### 4.2 Potential Ecological and Biogeochemical Implications of Protist Community Shift

The shift between the dominance of heterotroph and photoautotrophic functions during the stratified, oligotrophic season and the mesotrophic mixing season, provides insights into important transitions in ecological and biogeochemical processes in the GoA. Heterotrophic or photoautotrophic‐dominated food web structures differ fundamentally in energy flow and transfer efficiency to higher trophic levels, nutrient cycling and export production (Frederiksen et al. 2006). Typically, heterotrophic protists are important top‐down regulators and essential links in pelagic food webs, first as main consumers of primary production and subsequently as prey to larger zooplankton (Calbet 2008). The primary heterotrophic function detected during the oligotrophic season was parasites. These, like viruses, can reroute a portion of host biomass via host‐cell lysis away from the traditional food web and, therefore, supply nutrients to heterotrophic bacteria as part of the microbial loop (Thompson et al. 2005). It is also possible that other non‐host protistan heterotrophs eat part of parasitic cells (typically in the picoplankton size fractions) and directly link to higher trophic levels (Johnson et al. 2010). Moreover, phago‐mixotroph phytoplankton taxa from the phyla Dinoflagellata and Haptophyta were also common in the GoA during summer. These are the primary consumers of heterotrophic bacteria (Hartmann et al. 2012; Zubkov and Tarran 2008). Consequently, organic nutrients from bacteria can now support primary production (Hartmann et al. 2012; Mitra et al. 2014; Unrein et al. 2014). Hence, the coupled existence of parasites enhancing bacterial growth and phago‐mixotrophic phytoplankton that can consume these bacteria can act cooperatively, positively influencing each other via the microbial loop. These cooperative associations could further establish shorter and potentially more efficient links from nutrient regeneration to primary production, facilitating phytoplankton growth during oligotrophic periods. By contrast, during periods dominated by photoautotrophs, the establishment of a more classical food web structure from primary producers to higher trophic levels likely prevails. This food web structure is further exacerbated during the diatom bloom, as we detected a rapid increase of heterotrophic protists, which then could serve as food to larger zooplankton.

Finally, processes driving carbon export and nutrient recycling may differ under the dominance of heterotrophs (specifically parasites) and photoautotrophs. Recent work detected a strong negative correlation between Syndiniales and particulate organic carbon fluxes, suggesting that parasites attenuate the fluxes of carbon export and recycling (Anderson and Harvey 2020). By contrast, the build‐up of photoautotrophic biomass during winter and bloom comprising heavily‐armoured diatoms and coccolithophores can boost carbon export (Li et al. 2024; Tréguer et al. 2018). This scenario is consistent with local measurements of carbon export rates in the GoA, which are lower during summer and increase as a function of mixing depth and bloom size (Keuter et al. 2023; Torfstein et al. 2020).

### 4.3 Resemblance Between Protist Communities in the Stratified GoA and the Open Ocean

Similarities between the open sea in the GoA and other oligotrophic regions of the world ocean, are often evoked in the literature (Labiosa et al. 2003; Lindell and Post 1995; Reiss and Hottinger 1984). Here, by integrating our seasonal 18S rDNA dataset with the circum‐global *Tara* Oceans dataset (de Vargas et al. 2015), we confirm that during the stratified period, the planktonic protist assemblages from the GoA closely resemble assemblages from other warm, open ocean ecosystems worldwide. By contrast, assemblages observed during the bloom and, to some extent, in the DCM in the GoA were distinct. This uniqueness of winter communities in the GoA could result from the development of singular subtropical communities in response to unique oceanographic conditions in the GoA. However, it is plausible that future comparisons with amplicon sequencing datasets from coastal and upwelling regions that were absent from the *Tara* Oceans dataset will better enable us to relate the communities of protists found in the GoA during winter and the bloom to other global regions.

## 5 Conclusions

**FIGURE 7:**
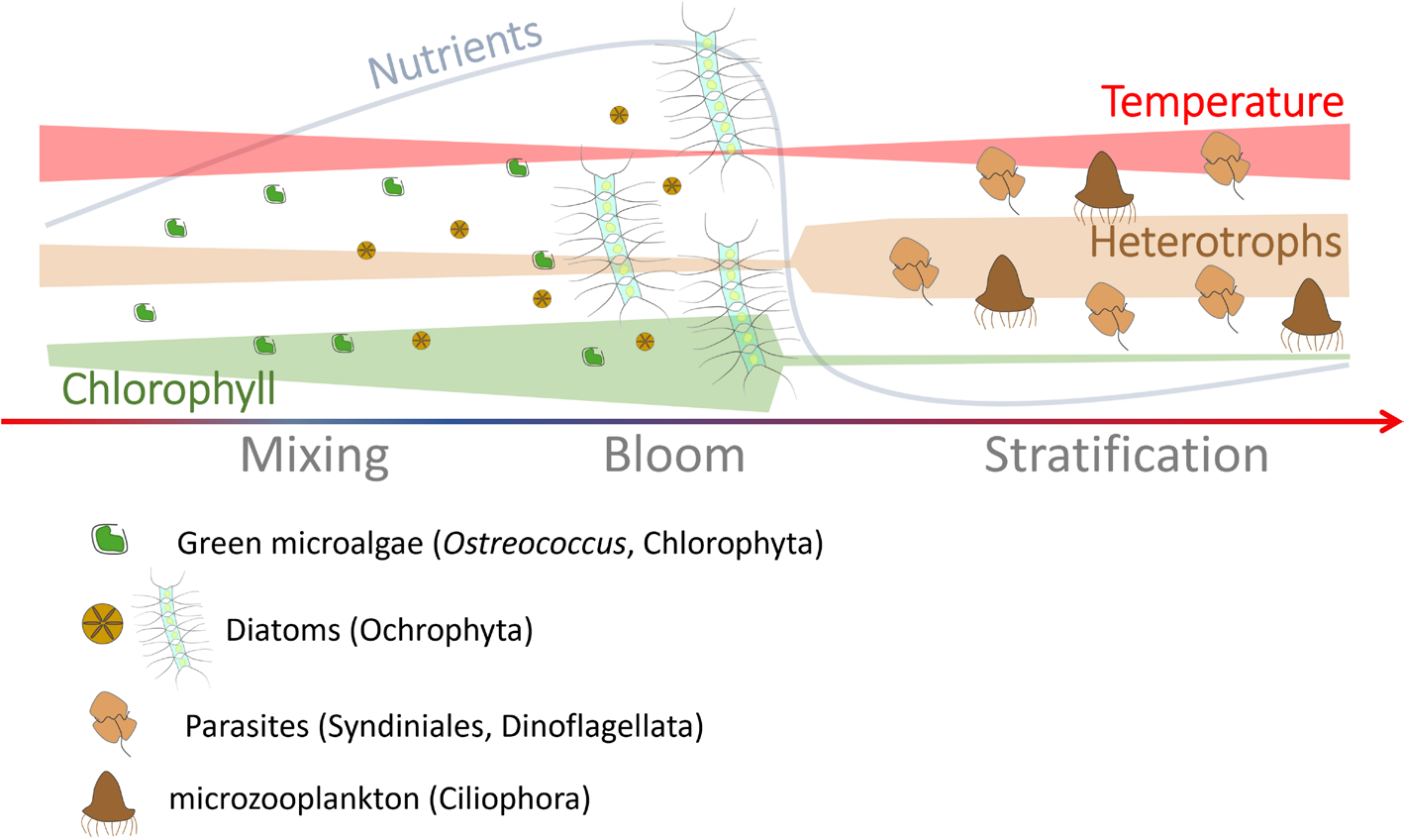
Diagrammatic portrayal of the changes to the planktonic Protits assemblages over a year in the GoA. It shows changing patterns of inorganic nutrients, temperature and the relative proportion of photoautotrophs and heterotrophs over the main seasons: The cold, mesotrophic winter‐mixing, the bloom at the onset of stratification and the warmer, stratified and oligotrophic summer. Some of the main taxa are depicted, particularly chlorophytes, diatoms as main phytoplankton more abundant during the winter and bloom, and dinoflagellates, parasitic Syndiniales and ciliates more common during the stratified season.

Here, we provide the first detailed synthesis of the diversity and seasonality of protist assemblages from the pico‐nano plankton in the epipelagic sunlit layer of the subtropical GoA. This seasonality involved a pronounced transition between the dominance of heterotrophic and autotrophic communities between the stratified and winter‐mixing and bloom seasons, respectively. A simple diagrammatic portrayal summarises the succession observed in the GoA (Figure 7). We further detected close similarities between planktonic assemblages from the GoA and other (sub)tropical regions from the world's open ocean, indicating that the stratified season in the GoA can serve as an open‐sea oligotrophic ecosystem model for long‐term monitoring, experimental studies and isolation of oligotrophic protist taxa. As such, our study adds to the current effort to better understand the ecology of oligotrophic marine ecosystems. In addition, the ecological succession presented here across oligotrophic‐mesotrophic gradients in the GoA can also represent a case study simulating the responses of protist communities to a shift in trophic regimes. Future quantification of the contribution of specific groups to primary production, functional traits and export fluxes, including parasites, will enable us to quantify better ecosystem‐level processes, and the response of protist communities to environmental changes in subtropical ecosystems.

## Author Contributions

**Yoav Avrahami:** conceptualization, data curation, formal analysis, investigation, methodology, software, validation, visualization, writing – original draft, writing – review and editing. **Raffaele Siano:** methodology, validation, writing – review and editing. **Maxim Rubin‐Blum:** methodology, validation, writing – review and editing. **Gil Koplovitz:** investigation, project administration. **Nicolas Henry:** methodology, validation. **Colomban de Vargas:** resources, writing – review and editing. **Miguel J. Frada:** conceptualization, formal analysis, funding acquisition, project administration, validation, supervision, writing – original draft, writing – review and editing.

## Conflicts of Interest

The authors declare no conflicts of interest.

## Supporting information


**Data S1:** Supporting Information.

**Table S1:** Supporting Information.

**Table S2:** Supporting Information.

## Data Availability

The raw 18S rRNA amplicon data for this study can befound in the National Centre for Biotechnology Information (NCBI) database under BioProject PRJNA1169359: https://www.ncbi.nlm.nih.gov/bioproject/?term=PRJNA1169359.

## References

[emi470126-bib-0001] Alberti, A. , J. Poulain , S. Engelen , et al. 2017. “Viral to Metazoan Marine Plankton Nucleotide Sequences From the Tara Oceans Expedition.” Scientific Data 4, no. 1: 1–20. 10.1038/sdata.2017.93. PMC5538240 28763055

[emi470126-bib-0002] Al‐Najjar, T. , M. I. Badran , C. Richter , M. Meyerhoefer , and U. Sommer . 2007. “Seasonal Dynamics of Phytoplankton in the Gulf of Aqaba, Red Sea.” Hydrobiologia 579, no. 1: 69–83. 10.1007/s10750-006-0365-z.

[emi470126-bib-0003] Anderson, S. R. , L. Blanco‐Bercial , C. A. Carlson , and E. L. Harvey . 2024. “Role of Syndiniales Parasites in Depth‐Specific Networks and Carbon Flux in the Oligotrophic Ocean.” ISME Communications 4, no. 1: 14. 10.1093/ismeco/ycae014. PMC10900894 38419659

[emi470126-bib-0004] Anderson, S. R. , and E. L. Harvey . 2020. “Temporal Variability and Ecological Interactions of Parasitic Marine Syndiniales in Coastal Protist Communities.” mSphere 5, no. 3: 10–1128. 10.1128/msphere.00209-20. PMC7253595 32461270

[emi470126-bib-0005] Avrahami, Y. , and M. J. Frada . 2020. “Detection of Phagotrophy in the Marine Phytoplankton Group of the Coccolithophores (Calcihaptophycidae, Haptophyta) During Nutrient‐Replete and Phosphate‐Limited Growth.” Journal of Phycology 56, no. 4: 1103–1108. 10.1111/jpy.12997. 32233088

[emi470126-bib-0006] Avrahami, Y. , G. Koplovitz , and M. J. Frada . 2025. “Diatom Community Succession and Bloom Variability as a Function of Winter‐Mixing Depth in the Subtropical Gulf of Aqaba, Red Sea.” Marine Ecology Progress Series 760: 39–54. 10.3354/meps14848.

[emi470126-bib-0007] Barlow, R. G. , R. F. C. Mantoura , M. A. Gough , and T. W. Fileman . 1993. “Pigment Signatures of the Phytoplankton Composition in the Northeastern Atlantic During the 1990 Spring Bloom.” Deep Sea Research Part II: Topical Studies in Oceanography 40, no. 1: 459–477.

[emi470126-bib-0008] Bass, D. , S. Rueckert , R. Stern , et al. 2021. “Parasites, Pathogens, and Other Symbionts of Copepods.” Trends in Parasitology 37, no. 10: 875–889. 10.1016/j.pt.2021.05.006. 34158247

[emi470126-bib-0009] Behrenfeld, M. J. , J. T. Randerson , C. R. McClain , et al. 2001. “Biospheric Primary Production During an ENSO Transition.” Science 291, no. 5513: 2594–2597. 11283369 10.1126/science.1055071

[emi470126-bib-0011] Blanco‐Bercial, L. , R. Parsons , L. M. Bolaños , R. Johnson , S. J. Giovannoni , and R. Curry . 2022. “The Protist Community Traces Seasonality and Mesoscale Hydrographic Features in the Oligotrophic Sargasso Sea.” Frontiers in Marine Science 9: 897140. 10.3389/fmars.2022.897140.

[emi470126-bib-0012] Blindheim, S. , L. Andersen , C. Trösse , E. Karlsbakk , and A. Nylund . 2023. “Growth Characteristics and Morphology of Paramoeba Perurans From Atlantic Salmon *Salmo salar* L. and Ballan Wrasse *Labrus bergylta* in Norway.” Parasites and Vectors 16, no. 1: 112. 10.1186/s13071-023-05715-2. 36959596 PMC10037839

[emi470126-bib-0013] Bolyen, E. , J. R. Rideout , M. R. Dillon , et al. 2019. “Reproducible, Interactive, Scalable and Extensible Microbiome Data Science Using QIIME 2.” Nature Biotechnology 37, no. 8: 852–857. 10.1038/s41587-019-0190-3. PMC7015180 31341288

[emi470126-bib-0014] Calbet, A. 2008. “The Trophic Roles of Microzooplankton in Marine Systems.” ICES Journal of Marine Science 65, no. 3: 325–331. https://academic.oup.com/icesjms/article/65/3/325/786057.

[emi470126-bib-0015] Callahan, B. J. , P. J. McMurdie , M. J. Rosen , A. W. Han , A. J. A. Johnson , and S. P. Holmes . 2016. “DADA2: High‐Resolution Sample Inference From Illumina Amplicon Data.” Nature Methods 13, no. 7: 581–583. 10.1038/nmeth.3869. 27214047 PMC4927377

[emi470126-bib-0016] Camacho, C. , G. Coulouris , V. Avagyan , et al. 2009. “BLAST+: Architecture and Applications.” BMC Bioinformatics 10: 1–9. 10.1186/1471-2105-10-421. 20003500 PMC2803857

[emi470126-bib-0017] Caron, D. A. , A. Z. Worden , P. D. Countway , E. Demir , and K. B. Heidelberg . 2009. “Protists Are Microbes Too: A Perspective.” ISME Journal 3, no. 1: 4–12. 10.1038/ismej.2008.101. 19005497

[emi470126-bib-0018] Cascella, A. , S. Bonomo , B. Jalali , et al. 2020. “Climate Variability of the Last ~2700 Years in the Southern Adriatic Sea: Coccolithophore Evidences.” Holocene 30, no. 1: 53–64. 10.1177/0959683619865600.

[emi470126-bib-0019] Chambouvet, A. , P. Morin , D. Marie , and L. Guillou . 2008. “Control of Toxic Marine Dinoflagellate Blooms by Serial Parasitic Killers.” Science 322, no. 5905: 1254–1257. 10.1126/science.1163971. 19023082

[emi470126-bib-0020] Cheng, L. , K. von Schuckmann , J. P. Abraham , et al. 2022. “Past and Future Ocean Warming.” Nature Reviews Earth and Environment 3, no. 11: 776–794. 10.1038/s43017-022-00345-1.

[emi470126-bib-0021] Choi, C. J. , V. Jimenez , D. M. Needham , et al. 2020. “Seasonal and Geographical Transitions in Eukaryotic Phytoplankton Community Structure in the Atlantic and Pacific Oceans.” Frontiers in Microbiology 11: 542372. 10.3389/fmicb.2020.542372. 33101224 PMC7554337

[emi470126-bib-0022] Christaki, U. , C. Courties , R. Massana , et al. 2011. “Optimized Routine Flow Cytometric Enumeration of Heterotrophic Flagellates Using SYBR Green I.” Limnology and Oceanography: Methods 9, no. 8: 329–339. 10.4319/lom.2011.9.329.

[emi470126-bib-0023] Christaki, U. , D. I. Skouroliakou , and L. Jardillier . 2023. “Interannual Dynamics of Putative Parasites (Syndiniales Group II) in a Coastal Ecosystem.” Environmental Microbiology 25, no. 7: 1314–1328. 10.1111/1462-2920.16358. 36852823

[emi470126-bib-0024] Chust, G. , J. I. Allen , L. Bopp , et al. 2014. “Biomass Changes and Trophic Amplification of Plankton in a Warmer Ocean.” Global Change Biology 20, no. 7: 2124–2139. 10.1111/gcb.12562. 24604761

[emi470126-bib-0025] Coats, D. W. 1999. “Parasitic Life Styles of Marine Dinoflagellates.” Journal of Eukaryotic Microbiology 46, no. 4: 402–409. 10.1111/j.1550-7408.1999.tb04620.x.

[emi470126-bib-0026] Coats, D. W. , and M. G. Park . 2002. “Parasitism of Photosynthetic Dinoflagellates by Three Strains of Amoebophrya (Dinophyta): Parasite Survival, Infectivity, Generation Time, and Host Specificity.” Journal of Phycology 38, no. 3: 520–528. 10.1046/j.1529-8817.2002.01200.x.

[emi470126-bib-0027] de Vargas, C. , S. Audic , N. Henry , et al. 2015. “Eukaryotic Plankton Diversity in the Sunlit Ocean.” Science 348, no. 6237: 1261605. https://www.science.org. 25999516 10.1126/science.1261605

[emi470126-bib-0028] Decelle, J. , E. Kayal , E. Bigeard , et al. 2022. “Intracellular Development and Impact of a Marine Eukaryotic Parasite on Its Zombified Microalgal Host.” ISME Journal 16, no. 10: 2348–2359. 10.1038/s41396-022-01274-z. 35804051 PMC9478091

[emi470126-bib-0029] Delage, E. , N. Henry , G. Salazar , et al. 2023. Tara Oceans (2009–2013) rDNA 18S V4 ASV Table (Dada2) [Data set].

[emi470126-bib-0030] Duarte, C. M. , A. Regaudie‐De‐Gioux , J. M. Arrieta , A. Delgado‐Huertas , and S. Agustí . 2013. “The Oligotrophic Ocean Is Heterotrophic.” Annual Review of Marine Science 5: 551–569. 10.1146/annurev-marine-121211-172337. 22809189

[emi470126-bib-0031] Eckmann, C. A. , C. Bachy , F. Wittmers , et al. 2024. “Recurring Seasonality Exposes Dominant Species and Niche Partitioning Strategies of Open Ocean Picoeukaryotic Algae.” Communications Earth & Environment 5, no. 1: 266. 10.1038/s43247-024-01395-7. 38779128 PMC11106004

[emi470126-bib-0032] Falkowski, P. G. , M. E. Katz , A. H. Knoll , et al. 2004. “The Evolution of Modern Eukaryotic Phytoplankton.” Science 305: 354–360. https://www.science.org. 15256663 10.1126/science.1095964

[emi470126-bib-0033] Finlay, B. J. 2004. “Protist Taxonomy: An Ecological Perspective.” Philosophical Transactions of the Royal Society of London. Series B, Biological Sciences 359, no. 1444: 599–610. 10.1098/rstb.2003.1450. 15253347 PMC1693346

[emi470126-bib-0034] Frada, M. J. , S. Keuter , G. Koplovitz , and Y. Avrahami . 2022. “Divergent Fate of Coccolithophores in a Warming Tropical Ecosystem.” Global Change Biology 28, no. 4: 1560–1568. 10.1111/gcb.16007. 34808010

[emi470126-bib-0035] Frederiksen, M. , M. Edwards , A. J. Richardson , N. C. Halliday , and S. Wanless . 2006. “From Plankton to Top Predators: Bottom‐Up Control of a Marine Food Web Across Four Trophic Levels.” Journal of Animal Ecology 75, no. 6: 1259–1268. 10.1111/j.1365-2656.2006.01148.x. 17032358

[emi470126-bib-0036] Genin, A. , B. Lazar , and S. Brenner . 1995. “Vertical Mixing and Coral Death in the Red Sea Following the Eruption of Mount Pinatubo.” Nature 377, no. 6549: 507–510.

[emi470126-bib-0037] Gómez, F. 2009. “ *Torodinium* and *Pavillardia* (Gymnodiniales, Dino‐Phyceae): Two Unarmoured Dinoflagellates With a Body Extension, Collected From the Open Pacific Ocean.” Protistology 6, no. 2: 131–135.

[emi470126-bib-0038] Gower, J. C. 1971. “A General Coefficient of Similarity and Some of Its Properties.” Biometrics 27, no. 4: 857–871. https://about.jstor.org/terms.

[emi470126-bib-0039] Grasshof, K. , K. Kremling , and M. Eharhardt . 2009. Methods of Seawater Analysis, edited by K. Grasshof , K. Kremling , and M. Eharhardt , 3rd ed. John Wiley & Sons.

[emi470126-bib-0040] Guidi, L. , S. Chaffron , L. Bittner , et al. 2016. “Plankton Networks Driving Carbon Export in the Oligotrophic Ocean.” Nature 532, no. 7600: 465–470. 10.1038/nature16942. 26863193 PMC4851848

[emi470126-bib-0041] Guillou, L. , D. Bachar , S. Audic , et al. 2013. “The Protist Ribosomal Reference Database (PR2): A Catalog of Unicellular Eukaryote Small Sub‐Unit rRNA Sequences With Curated Taxonomy.” Nucleic Acids Research 41, no. D1: D597–D604. 10.1093/nar/gks1160. 23193267 PMC3531120

[emi470126-bib-0042] Guillou, L. , M. Viprey , A. Chambouvet , et al. 2008. “Widespread Occurrence and Genetic Diversity of Marine Parasitoids Belonging to Syndiniales (Alveolata).” Environmental Microbiology 10, no. 12: 3349–3365. 10.1111/j.1462-2920.2008.01731.x. 18771501

[emi470126-bib-0043] Hallett, L. M. , S. K. Jones , A. A. M. MacDonald , et al. 2016. “Codyn: An r Package of Community Dynamics Metrics.” Methods in Ecology and Evolution 7, no. 10: 1146–1151. 10.1111/2041-210X.12569.

[emi470126-bib-0044] Hartmann, M. , C. Grob , G. A. Tarran , et al. 2012. “Mixotrophic Basis of Atlantic Oligotrophic Ecosystems.” Proceedings of the National Academy of Sciences of the United States of America 109, no. 15: 5756–5760. 10.1073/pnas.1118179109. 22451938 PMC3326507

[emi470126-bib-0045] James, C. C. , A. D. Barton , L. Z. Allen , et al. 2022. “Influence of Nutrient Supply on Plankton Microbiome Biodiversity and Distribution in a Coastal Upwelling Region.” Nature Communications 13, no. 1: 2448. 10.1038/s41467-022-30139-4. PMC9068609 35508497

[emi470126-bib-0046] Jeffrey, S. W. , and G. F. Humphrey . 1975. “New Spectrophotometric Equations for Determining Chlorophylls a, b, c1 and c2 in Higher Plants, Algae and Natural Phytoplankton.” Biochemie und Physiologie der Pflanzen 167, no. 2: 191–194.

[emi470126-bib-0047] Jeong, H. J. , Y. D. Yoo , K. H. Seong , et al. 2005. “Feeding by the Mixotrophic Red‐Tide Dinoflagellate *Gonyaulax Polygramma*: Mechanisms, Prey Species, Effects of Prey Concentration, and Grazing Impact.” Aquatic Microbial Ecology 38: 249–257.

[emi470126-bib-0048] Johnson, P. T. J. , A. Dobson , K. D. Lafferty , et al. 2010. “When Parasites Become Prey: Ecological and Epidemiological Significance of Eating Parasites.” Trends in Ecology & Evolution 25, no. 6: 362–371. 10.1016/j.tree.2010.01.005. 20185202

[emi470126-bib-0049] Keeling, P. J. 2013. “Elephants in the Room: Protists and He Importance of Morphology and Behaviour.” Environmental Microbiology Reports 5: 5–6. 10.1111/1758-2229.12021.

[emi470126-bib-0050] Keeling, P. J. , and J. del Campo . 2017. “Marine Protists Are Not Just Big Bacteria.” Current Biology 27, no. 11: R541–R549. 10.1016/j.cub.2017.03.075. 28586691

[emi470126-bib-0051] Keuter, S. , G. Koplovitz , A. Torfstein , and M. J. Frada . 2023. “Two‐Year Seasonality (2017, 2018), Export and Long‐Term Changes in Coccolithophore Communities in the Subtropical Ecosystem of the Gulf of Aqaba, Red Sea.” Deep‐Sea Research Part I: Oceanographic Research Papers 191: 103919. 10.1016/j.dsr.2022.103919.

[emi470126-bib-0052] Kranzler, C. F. , J. W. Krause , M. A. Brzezinski , et al. 2019. “Silicon Limitation Facilitates Virus Infection and Mortality of Marine Diatoms.” Nature Microbiology 4, no. 11: 1790–1797. 10.1038/s41564-019-0502-x. 31308524

[emi470126-bib-0053] Labiosa, R. G. , K. R. Arrigo , A. Genin , S. G. Monismith , and G. Van Dijken . 2003. “The Interplay Between Upwelling and Deep Convective Mixing in Determining the Seasonal Phytoplankton Dynamics in the Gulf of Aqaba: Evidence From SeaWiFS and MODIS.” Limnology and Oceanography 48, no. 6: 2355–2368. 10.4319/lo.2003.48.6.2355.

[emi470126-bib-0054] Lambert, B. S. , R. D. Groussman , M. J. Schatz , et al. 2022. “The Dynamic Trophic Architecture of Open‐Ocean Protist Communities Revealed Through Machine‐Guided Metatranscriptomics.” Proceedings of the National Academy of Sciences of the United States of America 119, no. 7: e2100916119. 10.1073/pnas.2100916119. 35145022 PMC8851463

[emi470126-bib-0055] Landou, E. , B. Lazar , J. LaRoche , K. Fennel , and I. Berman‐Frank . 2023. “Contribution of Photic and Aphotic N2 Fixation to Production in an Oligotrophic Sea.” Limnology and Oceanography 68, no. 3: 692–708. 10.1002/lno.12303.

[emi470126-bib-0056] Li, S. , J. Zhu , X. Jin , Y. Feng , N. Jiao , and W. Zhang . 2024. “Multifaceted Contribution of Coccolithophores to Ocean Carbon Export.” Ocean‐Land‐Atmosphere Research 3: 0049. 10.34133/olar.0049.

[emi470126-bib-0057] Lima‐Mendez, G. , K. Faust , N. Henry , et al. 2015. “Determinants of Community Structure in the Global Plankton Interactome.” Science 348, no. 6237: 1262073. 10.1126/science.1262073. 25999517

[emi470126-bib-0058] Lindell, D. , and A. F. Post . 1995. “Ultraphytoplankton Succession Is Triggered by Deep Winter Mixing in the Gulf of Aqaba (Eilat), Red Sea.” Limnology and Oceanography 40, no. 6: 1130–1141. 10.4319/lo.1995.40.6.1130.

[emi470126-bib-0059] Litchman, E. , C. A. Klausmeier , O. M. Schofield , and P. G. Falkowski . 2007. “The Role of Functional Traits and Trade‐Offs in Structuring Phytoplankton Communities: Scaling From Cellular to Ecosystem Level.” Ecology Letters 10, no. 12: 1170–1181. 10.1111/j.1461-0248.2007.01117.x. 17927770

[emi470126-bib-0060] Longhurst, A. 1995. “Seasonal Cycles of Pelagic Production and Consumption.” Progress in Oceanography 36, no. 2: 77–167.

[emi470126-bib-0061] Magneville, C. , N. Loiseau , C. Albouy , et al. 2022. “mFD: An R Package to Compute and Illustrate the Multiple Facets of Functional Diversity.” Ecography 2022, no. 1: e05904. 10.1111/ecog.05904.

[emi470126-bib-0062] Malviya, S. , E. Scalco , S. Audic , et al. 2016. “Insights Into Global Diatom Distribution and Diversity in the World's Ocean.” Proceedings of the National Academy of Sciences of the United States of America 113, no. 11: E1516–E1525. 10.1073/pnas.1509523113. 26929361 PMC4801293

[emi470126-bib-0063] Marie, D. , F. Partensky , D. Vaulot , and C. Brussard . 1999. “Enumeration of Phytoplankton, Bacteria, and Viruses in Marine Samples.” Current Protocols in Cytometry 10, no. 1: 11. 10.1002/0471142956.cy1111s10 18770685

[emi470126-bib-0064] Martin, M. 2011. “Cutadapt Removes Adapter Sequences From High‐Throughput Sequencing Reads.” EMBnet Journal 17, no. 1: 10–12. http://www‐huber.embl.de/users/an‐.

[emi470126-bib-0065] Massana, R. , J. Del Campo , M. E. Sieracki , S. Audic , and R. Logares . 2014. “Exploring the Uncultured Microeukaryote Majority in the Oceans: Reevaluation of Ribogroups Within Stramenopiles.” ISME Journal 8, no. 4: 854–866. 10.1038/ismej.2013.204. 24196325 PMC3960543

[emi470126-bib-0066] Massana, R. , A. Gobet , S. Audic , et al. 2015. “Marine Protist Diversity in European Coastal Waters and Sediments as Revealed by High‐Throughput Sequencing.” Environmental Microbiology 17, no. 10: 4035–4049. 10.1111/1462-2920.12955. 26119494

[emi470126-bib-0067] McMurdie, P. J. , and S. Holmes . 2013. “Phyloseq: An R Package for Reproducible Interactive Analysis and Graphics of Microbiome Census Data.” PLoS One 8, no. 4: 61217. 10.1371/journal.pone.0061217. PMC3632530 23630581

[emi470126-bib-0068] Meyneng, M. , H. Lemonnier , R. Le Gendre , et al. 2024. “Subtropical Coastal Microbiome Variations due to Massive River Runoff After a Cyclonic Event.” Environmental Microbiomes 19, no. 1: 10. 10.1186/s40793-024-00554-9. PMC10829310 38291506

[emi470126-bib-0069] Mitra, A. , D. A. Caron , E. Faure , et al. 2023. “The Mixoplankton Database (MDB): Diversity of Photo‐Phago‐Trophic Plankton in Form, Function, and Distribution Across the Global Ocean.” Journal of Eukaryotic Microbiology 70, no. 4: e12972. 10.1111/jeu.12972. 36847544

[emi470126-bib-0070] Mitra, A. , K. J. Flynn , J. M. Burkholder , et al. 2014. “The Role of Mixotrophic Protists in the Biological Carbon Pump.” Biogeosciences 11, no. 4: 995–1005. 10.5194/bg-11-995-2014.

[emi470126-bib-0071] Nagarkar, M. , and B. Palenik . 2023. “Diversity and Putative Interactions of Parasitic Alveolates Belonging to Syndiniales at a Coastal Pacific Site.” Environmental Microbiology Reports 15, no. 3: 157–169. 10.1111/1758-2229.13138. 36779254 PMC10464665

[emi470126-bib-0072] Oksanen, J. , G. L. Simpson , G. F. Blanchet , et al. 2022. “Vegan: Community Ecology Package.” (2.6–4). R Package. http://vegan.r‐forge.r‐project.org/.

[emi470126-bib-0073] Ollison, G. A. , S. K. Hu , L. Y. Mesrop , E. F. DeLong , and D. A. Caron . 2021. “Come Rain or Shine: Depth Not Season Shapes the Active Protistan Community at Station ALOHA in the North Pacific Subtropical Gyre.” Deep‐Sea Research Part I: Oceanographic Research Papers 170: 103494. 10.1016/j.dsr.2021.103494.

[emi470126-bib-0074] Pasulka, A. L. , M. R. Landry , D. A. A. Taniguchi , A. G. Taylor , and M. J. Church . 2013. “Temporal Dynamics of Phytoplankton and Heterotrophic Protists at Station ALOHA.” Deep‐Sea Research Part II: Topical Studies in Oceanography 93: 44–57. 10.1016/j.dsr2.2013.01.007.

[emi470126-bib-0075] Pernice, M. C. , C. R. Giner , R. Logares , et al. 2016. “Large Variability of Bathypelagic Microbial Eukaryotic Communities Across the World's Oceans.” ISME Journal 10, no. 4: 945–958. 10.1038/ismej.2015.170. 26451501 PMC4796934

[emi470126-bib-0076] Pesant, S. , F. Not , M. Picheral , et al. 2015. “Open Science Resources for the Discovery and Analysis of Tara Oceans Data.” Scientific Data 2, no. 1: 1–16. 10.1038/sdata.2015.23. PMC4443879 26029378

[emi470126-bib-0077] Polovina, J. J. , E. A. Howell , and M. Abecassis . 2008. “Ocean's Least Productive Waters Are Expanding.” Geophysical Research Letters 35, no. 3: L03618. 10.1029/2007GL031745.

[emi470126-bib-0078] R Core Team . 2021. R: A Language and Environment for Statistical Computing. R Foundation for Statistical Computing (4.2.1). https://www.r‐project.org/.

[emi470126-bib-0079] Ramond, P. , P. E. Galand , and R. Logares . 2024. “Microbial Functional Diversity and Redundancy: Moving Forward.” FEMS Microbiology Reviews 49: fuae031. 10.1093/femsre/fuae031. PMC11756291 39689915

[emi470126-bib-0080] Ramond, P. , M. Sourisseau , N. Simon , et al. 2019. “Coupling Between Taxonomic and Functional Diversity in Protistan Coastal Communities.” Environmental Microbiology 21, no. 2: 730–749. 10.1111/1462-2920.14537. 30672084

[emi470126-bib-0082] Reiss, Z. , and L. Hottinger . 1984. “A Desert‐Enclosed Sea.” In The Gulf of Aqaba, vol. 50, 33–88. Springer Berlin Heidelberg. 10.1007/978-3-642-69787-6.

[emi470126-bib-0083] Rizos, I. , P. Debeljak , T. Finet , et al. 2023. “Beyond the Limits of the Unassigned Protist Microbiome: Inferring Large‐Scale Spatio‐Temporal Patterns of Syndiniales Marine Parasites.” ISME Communications 3, no. 1: 16. 10.1038/s43705-022-00203-7. 36854980 PMC9975217

[emi470126-bib-0084] Santoferrara, L. F. 2019. “Current Practice in Plankton Metabarcoding: Optimization and Error Management.” Journal of Plankton Research 41, no. 5: 571–582. 10.1093/plankt/fbz041.

[emi470126-bib-0085] Shaked, Y. , and M. Fine . 2023. The Israel National Monitoring Program at the Gulf of Eilat (NMP).

[emi470126-bib-0086] Sherr, E. B. , and B. F. Sherr . 2002. “Significance of Predation by Protists in Aquatic Microbial Food Webs.” Antonie Van Leeuwenhoek 81: 293–308. 10.1023/A:1020591307260. 12448728

[emi470126-bib-0087] Siano, R. , C. Alves‐de‐Souza , E. Foulon , et al. 2011. “Distribution and Host Diversity of Amoebophryidae Parasites Across Oligotrophic Waters of the Mediterranean Sea.” Biogeosciences 8, no. 2: 267–278. 10.5194/bg-8-267-2011.

[emi470126-bib-0088] Smalley, G. W. , D. W. Coats , and D. K. Stoecker . 2003. “Feeding in the Mixotrophic Dinoflagellate *Ceratium furca* Is Influenced by Intracellular Nutrient Concentrations.” Marine Ecology Progress Series 262: 137–151.

[emi470126-bib-0089] Stoeck, T. , D. Bass , M. Nebel , et al. 2010. “Multiple Marker Parallel Tag Environmental DNA Sequencing Reveals a Highly Complex Eukaryotic Community in Marine Anoxic Water.” Molecular Ecology 19: 21–31. 10.1111/j.1365-294X.2009.04480.x. 20331767

[emi470126-bib-0090] Stoecker, D. K. , P. J. Hansen , D. A. Caron , and A. Mitra . 2017. “Mixotrophy in the Marine Plankton.” Annual Review of Marine Science 9, no. 1: 311–335. 10.1146/annurev-marine-010816-060617. 27483121

[emi470126-bib-0091] Strickland, J. D. H. , and T. R. Parsons . 1972. A Practical Handbook of Seawater Analysis, edited by J. C. Stevenson , 2nd ed. Bulletin of Fisheries Research.

[emi470126-bib-0092] Thompson, R. M. , K. N. Mouritsen , and R. Poulin . 2005. “Importance of Parasites and Their Life Cycle Characteristics in Determining the Structure of a Large Marine Food Web.” Journal of Animal Ecology 74, no. 1: 77–85. 10.1111/j.1365-2656.2004.00899.x.

[emi470126-bib-0093] Torfstein, A. , S. S. Kienast , B. Yarden , A. Rivlin , S. Isaacs , and Y. Shaked . 2020. “Bulk and Export Production Fluxes in the Gulf of Aqaba, Northern Red Sea.” ACS Earth and Space Chemistry 4, no. 8: 1461–1479. 10.1021/acsearthspacechem.0c00079.

[emi470126-bib-0094] Tréguer, P. , C. Bowler , B. Moriceau , et al. 2018. “Influence of Diatom Diversity on the Ocean Biological Carbon Pump.” Nature Geoscience 11, no. 1: 27–37. 10.1038/s41561-017-0028-x.

[emi470126-bib-0095] Treusch, A. H. , E. Demir‐Hilton , K. L. Vergin , et al. 2012. “Phytoplankton Distribution Patterns in the Northwestern Sargasso Sea Revealed by Small Subunit rRNA Genes From Plastids.” ISME Journal 6, no. 3: 481–492. 10.1038/ismej.2011.117. 21955994 PMC3280133

[emi470126-bib-0096] Unrein, F. , J. M. Gasol , F. Not , I. Forn , and R. Massana . 2014. “Mixotrophic Haptophytes Are Key Bacterial Grazers in Oligotrophic Coastal Waters.” ISME Journal 8, no. 1: 164–176. 10.1038/ismej.2013.132. 23924785 PMC3869011

[emi470126-bib-0097] Villéger, S. , N. W. H. Mason , and D. Mouillot . 2008. “New Multidimensional Functional Diversity Indices for a Multifaceted Framework in Functional Ecology.” Ecology 89, no. 8: 2290–2301. 10.1890/07-1206.1. 18724739

[emi470126-bib-0098] Violle, C. , D. R. Nemergut , Z. Pu , and L. Jiang . 2011. “Phylogenetic Limiting Similarity and Competitive Exclusion.” Ecology Letters 14, no. 8: 782–787. 10.1111/j.1461-0248.2011.01644.x. 21672121

[emi470126-bib-0099] Worden, A. Z. , M. J. Follows , S. J. Giovannoni , S. Wilken , A. E. Zimmerman , and P. J. Keeling . 2015. “Rethinking the Marine Carbon Cycle: Factoring in the Multifarious Lifestyles of Microbes.” Science 347, no. 6223: 1257594. 10.1126/science.1257594. 25678667

[emi470126-bib-0100] Yoo, Y. D. , K. A. Seong , H. J. Jeong , et al. 2017. “Mixotrophy in the Marine Red‐Tide Cryptophyte Teleaulax Amphioxeia and Ingestion and Grazing Impact of Cryptophytes on Natural Populations of Bacteria in Korean Coastal Waters.” Harmful Algae 68: 105–117. 10.1016/j.hal.2017.07.012. 28962973

[emi470126-bib-0101] Zamora‐Terol, S. , A. Novotny , and M. Winder . 2020. “Reconstructing Marine Plankton Food Web Interactions Using DNA Metabarcoding.” Molecular Ecology 29, no. 17: 3380–3395. 10.1111/mec.15555. 32681684

[emi470126-bib-0102] Zarubin, M. , Y. Lindemann , and A. Genin . 2017. “The Dispersion‐Confinement Mechanism: Phytoplankton Dynamics and the Spring Bloom in a Deeply‐Mixing Subtropical Sea.” Progress in Oceanography 155: 13–27. 10.1016/j.pocean.2017.05.005.

[emi470126-bib-0103] Zubkov, M. V. , and G. A. Tarran . 2008. “High Bacterivory by the Smallest Phytoplankton in the North Atlantic Ocean.” Nature 455, no. 7210: 224–226. 10.1038/nature07236. 18690208

